# Clinical validation of comprehensive time- and frequency-domain photoplethysmography features from a single-sensor system for non-invasive assessment of vascular load and systolic blood pressure

**DOI:** 10.3389/fphys.2025.1695391

**Published:** 2025-10-29

**Authors:** Chin-Nan Lin, Chih-Ping Chang, Jen-Jyh Lin, Jia-Ning Chang, Yen-Ching Hung, Po-Yen Ko

**Affiliations:** ^1^ China Medical University, Taichung, Taiwan; ^2^ Department of Medicine, China Medical University Hospital, Taichung, Taiwan; ^3^ Department of Bioinformatics and Medical Engineering, Asia University, Taichung, Taiwan

**Keywords:** photoplethysmography, systolic blood pressure, waveform sharpness, harmonic ratio, vascular stiffness, non-invasive screening

## Abstract

**Objective:**

Hypertension remains a silent yet powerful driver of cardiovascular disease—one of the world’s leading causes of death. Despite being treatable, it often goes undetected until complications arise. Photoplethysmography (PPG), a low-cost, non-invasive tool, holds promise for identifying vascular changes linked to high blood pressure. However, current single-site methods primarily focus on time-domain signals, often missing rich spectral data that could enhance early detection of hypertensive changes.

**Methods:**

We designed a compact, noise-resistant PPG acquisition system (Taiwan Patent No. 114206342) aimed at real-world usability and recruited 590 adults from cardiology outpatient clinics. Using a validated oscillometric device, systolic blood pressure (SBP) was measured and categorized into four groups (≤120, 121–139, 140–159, ≥160 mmHg). Ninety-second fingertip PPG recordings (500 Hz) underwent careful pre-processing—filtering, normalization, and artifact rejection. We extracted 19 features covering waveform shape, sharpness, heart rate variability (HRV), and frequency-domain harmonic ratios. Group differences were assessed using ANOVA or Kruskal–Wallis tests, with *post hoc* analyses and effect size reporting (epsilon squared, ε^2^).

**Results:**

Several features demonstrated strong associations with SBP levels. Waveform sharpness indices (1_10, 1_8, 1_6, 1_5, 1_3, 1_2) and harmonic ratios (H2/H1, H3/H1, H4/H1) showed a progressive decline as SBP increased—signaling a loss of higher-frequency components likely due to vascular stiffening. Time-domain features revealed that individuals with elevated SBP had prolonged systolic phases, shorter diastolic intervals, and higher Ts/Td ratios. Peak amplitudes (P1, P2), systolic and diastolic slopes, and overall waveform area also differed significantly by group. Effect sizes ranged from small to moderate, with the most pronounced changes seen in waveform sharpness.

**Conclusion:**

Our results demonstrate the promise of a single-sensor PPG-based approach for monitoring hypertension-related, subtle vascular alterations by combining morphological and spectral information. Progressive decline in harmonic ratios provides new information on arterial stiffening and changed wave reflections. This level of physiological depth combined with a remarkably simple and portable approach is the key that could allow wearable devices to provide immediate blood pressure screening for unprecedented widespread user engagement. These aspects are facilitated by making the test easier to operate than standard PCR and combining it with types of analyte detection that expand its performance capabilities in this specific disease context.

## 1 Introduction

Hypertension is one of the most pressing global health threats, affecting an estimated 1.4 billion people—over 31% of the world’s population in 2010 (Mills et al., 2016). Despite its widespread impact, fewer than half of those affected receive proper diagnosis or treatment, and nearly two-thirds are unaware of their condition (Egan et al., 2019). This silent epidemic carries heavy consequences: it is a key contributor to cardiovascular disease, stroke, chronic kidney disease, and other life-altering conditions (Lawes et al., 2008). Large-scale clinical trials confirm that early and effective blood pressure control—through lifestyle changes or medication—can significantly lower cardiovascular risk. Yet identifying those at risk before damage occurs remains a major public health challenge (Blood Pressure Lowering Treatment Trialists, 2021).

Photoplethysmography (PPG), a non-invasive optical technique that tracks pulsatile blood volume changes in the microvascular bed, offers an appealing path forward (Pankaj et al., 2022). Its affordability, simplicity, and compatibility with mobile or wearable devices make it ideal for expanding access to cardiovascular screening. Traditional PPG-based approaches, such as pulse transit time (PTT) and pulse wave velocity (PWV), are physiologically sound but often rely on dual-sensor configurations (e.g., ECG + PPG) and repeated calibration to accommodate individual differences (Esmaelpoor et al., 2020). These complexities limit scalability and hinder integration into everyday health monitoring.

Single-site pulse wave analysis (PWA) addresses this issue by extracting timing and shape features directly from a single PPG signal, reducing the need for multiple sensors. This simplification lowers technical barriers for integration into consumer electronics and broadens the scope for community-based screening. However, most current models focus exclusively on time-domain features, overlooking frequency-domain data that could capture deeper insights into vascular health.

In this study, we introduce a noise-resistant, single-sensor PPG system (Taiwan Patent No. 114206342) that blends time-domain PWA with Fourier-based spectral analysis to track both morphological and frequency-based changes linked to elevated systolic blood pressure (SBP). We place special emphasis on harmonic amplitude ratios, which reflect the energy of higher-order waveform components—features associated with arterial stiffening, altered wave reflections, and loss of dicrotic notches in hypertensive patients. By establishing clear links between these spectral markers and SBP categories in a real-world clinical sample, we demonstrate the practical potential of this low-cost approach. Our findings support its use in both clinical and consumer environments, offering a meaningful bridge between physiological research and scalable hypertension screening.

## 2 Materials and methods

### 2.1 Study design and participants

This cross-sectional study was conducted at China Medical University Hospital, Taichung, Taiwan. Participants were consecutively recruited from the cardiology outpatient clinics. Eligible participants were adults aged 20 years or older who were able to provide written informed consent and had been in a resting state for at least 10 min prior to measurement.

Exclusion criteria were as follows: (1) documented arrhythmias, such as atrial fibrillation; (2) presence of a permanent pacemaker; (3) inter-measurement interval <15 s; (4) undergoing maintenance dialysis; (5) severe peripheral vascular disease; (6) structural deformities of the fingers or wrists that could interfere with sensor placement; and (7) incomplete or excessively noisy photoplethysmographic (PPG) signals after preprocessing.

The study protocol was reviewed and approved by the Research Ethics Committee III of China Medical University and Hospital (Approval No. CMUH114-REC3-111). Written informed consent was obtained from all participants prior to enrollment.

Clinical and demographic information, including comorbidities (DM, CAD, CHF, CKD, thyroid disease, COPD/asthma) and laboratory data (LVEF, eGFR, LDL, statin use), were retrospectively obtained from participants’ electronic medical records.

**FIGURE 1:**
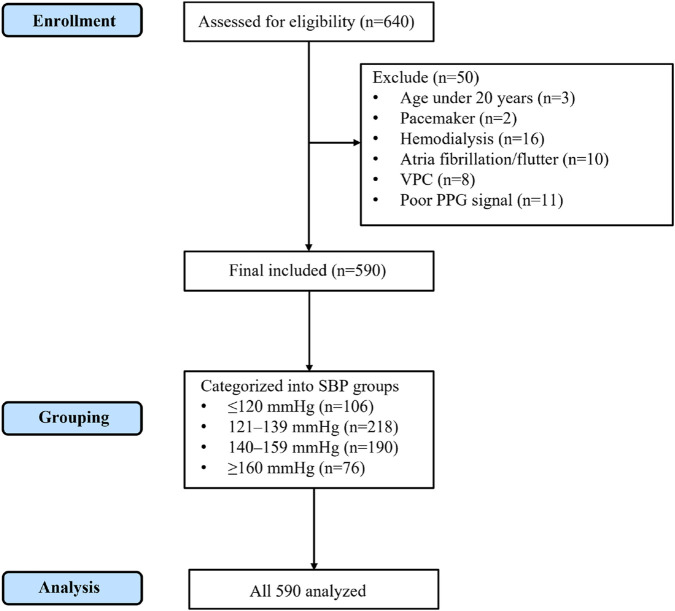
CONSORT flow diagram of participant enrollment, exclusion, grouping, and analysis. A total of 640 individuals were assessed for eligibility. After excluding 50 participants (3 aged under 20 years, 2 with pacemaker, 16 on hemodialysis, 10 with atrial fibrillation/flutter, 8 with frequent ventricular premature contractions, and 11 with poor PPG signal quality), 590 participants were included in the final analysis. These were subsequently categorized into four SBP groups (≤120 mmHg, 121–139 mmHg, 140–159 mmHg, and ≥160 mmHg), and all 590 participants were analyzed.

The process of participant enrollment, exclusion, and final analysis is shown in Figure 1 (CONSORT flow diagram).

### 2.2 Blood pressure measurement and grouping

Blood pressure (BP) was measured using a validated automated oscillometric sphygmomanometer (Model: HBP-9030, Manufacturer: Omron Healthcare Co., Ltd., Kyoto, Japan). Participants were seated comfortably with the right arm supported at heart level and rested for at least 10 min before measurement. Two BP readings were obtained at 1-min intervals, and the mean values of systolic BP (SBP) and diastolic BP (DBP) were recorded for analysis.

For subsequent analyses, participants were categorized into four systolic blood pressure (SBP in mmHg) groups in accordance with established clinical thresholds:

1. Group 1: SBP ≤120
2. Group 2: 120 < SBP ≤139
3. Group 3: 140 ≤ SBP ≤159
4. Group 4: SBP ≥160

This categorization followed the Seventh Report of the Joint National Committee (JNC 7) on Prevention, Detection, Evaluation, and Treatment of High Blood Pressure, which defines Normal (<120/80 mmHg), Prehypertension (120–139/80–89 mmHg), Stage 1 Hypertension (140–159/90–99 mmHg), and Stage 2 Hypertension (≥160/100 mmHg). Because arterial waveforms primarily reflect hemodynamic changes generated by ventricular ejection during systole, SBP values were used to define blood pressure categories in this study.

### 2.3 PPG signal acquisition and preprocessing

Photoplethysmographic (PPG) signals were acquired using a MAX30102 optical sensor (Maxim Integrated, USA) interfaced with a Raspberry Pi 5 (Raspberry Pi Foundation, UK) via the I^2^C communication protocol. The sampling rate was set to 500 Hz, and continuous recordings were obtained for 90 s per participant. During acquisition, the sensor was placed on the left index fingertip, with ambient light minimized to reduce optical interference.

Raw PPG signals underwent the following preprocessing procedures to ensure high signal fidelity:

- High-pass filtering (cutoff frequency: 0.5 Hz) to remove DC baseline drift.
- Low-pass filtering (cutoff frequency: 8 Hz) to suppress high-frequency noise.
- Amplitude normalization to a unit scale for inter-subject comparability.

Manual artifact rejection via visual inspection, excluding segments with motion artifacts or unstable sensor contact. Only clean, artifact-free waveforms were retained for subsequent feature extraction and statistical analysis.

The PPG acquisition device used in this study incorporates a proprietary noise-suppression and signal-stabilization design, which has been granted a Taiwan utility model patent (Patent No. 114206342). This technology enables stable waveform acquisition under typical clinical and ambient conditions. In terms of detection of the peak in PRV analysis, several fiducial points of the PPG waveform have been proposed in the literature. The most common techniques are: (1) the peak systolic, defined as the local maximum of each pulse cycle (Elgendi et al., 2013); (2) the onset (or foot) of the pulse, which is the inflection point at the beginning of the upstroke and frequently calculated with the second derivative of the PPG signal (Takazawa et al., 1998); and (3) the point of maximum slope, which is the point of steepest rise during the upstroke of systole, typically identified through the first derivative (Elgendi et al., 2013). Each fiducial definition has specific strengths and shortcomings depending on signal quality, application environment, and desired physiological interpretation. There is no current consensus regarding which point best offers beat-to-beat interval estimates, so peak detection remains a methodologically problematic issue in PRV research (Park et al., 2021).

We employed a systolic peak definition in this study, where a cardiac cycle was identified as the identification of the local maximum of the PPG signal. To ensure that we had correct peak localization, we applied a smoothing filter and subsequently applied zero-crossing detection to the first derivative, identifying points where the slope changes from positive to negative after the upstroke. This method has become widespread use in PPG-based analyses due to being computationally efficient, robust to minimal noise, and highly sensitive to signal detection at resting conditions (Suboh et al., 2022).

As all of our measurements were made under standard outpatient examination conditions, in which the subjects were rested and motionless in the sitting position, motion artifacts were insignificant. Under such controlled conditions, systolic peak was an effective and physiologically relevant fiducial point. On this basis, beat-to-beat intervals were thus computed as time differences between consecutive systolic peaks, and formed the platform for PRV calculations.

### 2.4 Feature extraction from PPG

To comprehensively characterize the pulse waveform and investigate its relationship with blood pressure, we extracted a total of 19 features from each 90-s PPG recording, comprising both time-domain and frequency-domain parameters (Table 1).

**TABLE 1:** Summary of photoplethysmography (PPG)-derived features used in the present study.

Feature	Domain	Description
Pulse rate	Time	Beats per minute
Ts	Time	Systolic duration
Td	Time	Diastolic duration
Ts/Td	Time	Ratio of systolic to diastolic duration
Ss	Time	Slopes of systolic phase
Ds	Time	Slopes of diastolic phase
Area	Time	Area under the pulse waveform
P1	Time	Peak after baseline correction
P2	Time	Reflected wave peak position after the dicrotic notch, computed after baseline correction
Delta_T	Time	Time interval between the occurrence of P1 and P2
SDNN	Time	Standard deviation of beat-to-beat intervals
RMSSD	Time	Root mean square of successive NN interval differences (vagal tone)
PNN50	Time	Percentage of NN interval differences >50 m
H1	Frequency	Main harmonic power from PPG waveform FFT
H2	Frequency	Secondary harmonic power from PPG waveform FFT
H3	Frequency	Third harmonic power from PPG waveform FFT
H4	Frequency	Fourth harmonic power from PPG waveform FFT
LF	Frequency	Power of interpolated PRV in low frequency bands
HF	Frequency	Power of interpolated PRV in high frequency bands

**FIGURE 2:**
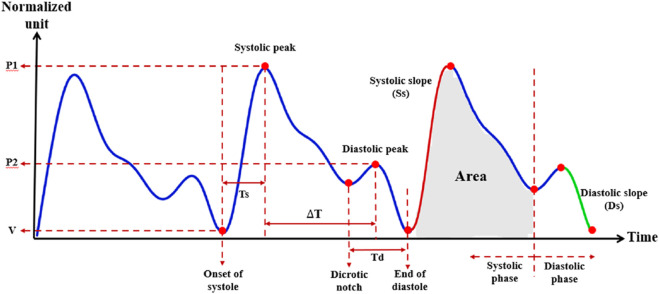
Photoplethysmography (PPG) waveform and extracted morphological parameters. Representative normalized PPG waveform illustrating key fiducial points and derived features used in the present study. Parameters include systolic peak amplitude (P1), diastolic peak amplitude (P2), systolic time (Ts), diastolic time (Td), time difference between systolic and diastolic peaks (ΔT), systolic slope (Ss), diastolic slope (Ds), and total area under the curve during the systolic phase. V indicates the preceding valley. The dicrotic notch marks the transition between systolic and diastolic phases.

Figure 2 presents a representative PPG waveform segment obtained from our measurement system, with key morphological landmarks and temporal intervals annotated. The diagram illustrates the systolic peak (P1), diastolic peak (P2), valley points (V), and the dicrotic notch, as well as derived parameters such as systolic time (Ts), diastolic time (Td), peak time difference (ΔT), systolic slope (Ss), diastolic slope (Ds), and the pulse area. These annotated features, derived directly from actual PPG recordings, serve as the basis for calculating morphological, amplitude-based, and timing indices in the time domain.

**FIGURE 3:**
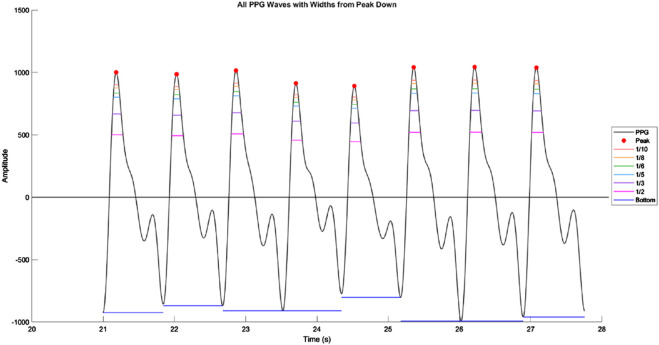
Illustration of waveform sharpness measurement in a photoplethysmography (PPG) signal. Representative segment of the PPG waveform showing multiple consecutive beats, with systolic peaks marked in red. Horizontal colored lines indicate the waveform widths at fractional amplitudes of the systolic peak (1/10, 1/8, 1/6, 1/5, 1/3, and 1/2 of peak height). These widths are measured from the left to the right intersection of the waveform with each target amplitude level, and normalized by the total beat duration to obtain sharpness ratios. The bottom horizontal line denotes the beat duration measured from valley to valley.

In addition to these conventional morphological features, we also calculated a set of waveform sharpness parameters (1_10, 1_8, 1_6, 1_5, 1_3, and 1_2). As illustrated in Figure 3, which is derived from an actual PPG waveform recorded by our measurement system, each parameter was defined by first identifying the target height:

$$Targetheight=P1-\frac{1}{n}\times P1$$

where n is 10, 8, 6, 5, 3, or 2, and P1 is the amplitude of the systolic peak relative to the baseline. The horizontal width at this target height was then determined by measuring the time difference between the left and right intersections of the waveform with the target height level. This width was normalized by the total beat duration, defined as the time from the preceding valley to the subsequent valley, to yield the final sharpness ratio.

#### 2.4.1 Time-domain features

The following 13 Time-Domain features were extracted from individual pulse waveforms to reflect morphological, temporal, and rhythmic characteristics:

Primary Peak Amplitude (P1): Amplitude of the main (first) systolic peak relative to the baseline.

Secondary Peak Amplitude (P2): Amplitude of the reflected (second) diastolic peak relative to the baseline.

Peak Time Difference (ΔT): Time interval between the occurrence of P1 and P2.

Systolic Time (Ts): Duration from the onset of the pulse to the systolic peak.

Diastolic Time (Td): Duration from the systolic peak to the end of the pulse.

Systolic Slope (Ss): Slope of the waveform during the systolic upstroke.

Diastolic Slope (Ds): Slope during the diastolic downstroke.

Pulse Area (Area): Total area under each pulse waveform.

Systole-to-Diastole Ratio (Ts/Td): Ratio of systolic to diastolic duration.

SDNN: Standard deviation of NN (beat-to-beat) intervals.

RMSSD: Root mean square of successive differences of NN intervals.

PNN50: Proportion of NN interval differences >50 m.

Pulse Rate: Beats per minute calculated from pulse peaks.

All above features were extracted on a beat-to-beat basis and then averaged across the 60-s segment.

#### 2.4.2 Frequency-domain features

Two types of frequency-domain features were derived: harmonic energy indicators and autonomic-related variability metrics.

**FIGURE 4:**
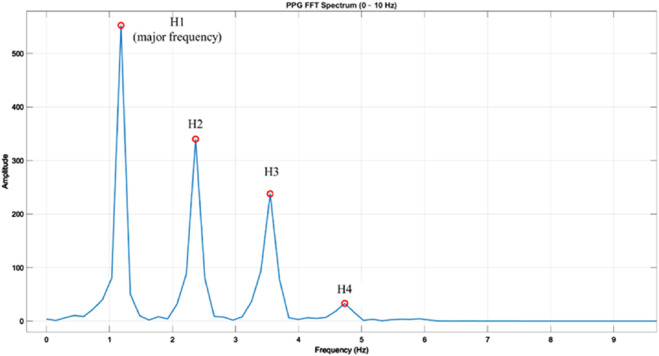
Frequency spectrum of a photoplethysmography (PPG) waveform obtained via fast Fourier transform (FFT). Example PPG beat transformed into the frequency domain using FFT, showing the fundamental frequency (H1) and higher-order harmonics (H2, H3, H4) within the 0–10 Hz range. H1 represents the major frequency component corresponding to the heart rate, while H2–H4 reflect harmonic contributions that characterize waveform morphology. Amplitudes are shown in arbitrary units, and harmonic ratios (Hk/H1) were later calculated to normalize inter-subject differences in signal magnitude.

Figure 4 shows a representative harmonic spectrum obtained by applying the Fast Fourier Transform (FFT) to a clean PPG waveform segment from our measurement system. The fundamental frequency (H1) corresponds to the dominant heart rate component, while higher-order harmonics (H2–H4) capture waveform sharpness and reflective wave contributions. The amplitudes of these harmonics, as well as their relative ratios (e.g., H2/H1, H3/H1, H4/H1), were extracted as quantitative descriptors of the spectral composition of the PPG signal. These harmonic ratios are sensitive to changes in arterial stiffness and wave reflection timing, thus providing complementary information to time-domain morphological parameters.

In addition, frequency-domain analysis of beat-to-beat interval series derived from PPG was performed to calculate autonomic-related variability metrics. Using Welch’s method, the power within the low-frequency (LF: 0.04–0.15 Hz) and high-frequency (HF: 0.15–0.40 Hz) bands was computed, and the LF/HF ratio was used as an index of sympathetic–parasympathetic balance.

1. Harmonic Components (H1–H4)

Each 90-s PPG segment was zero-padded and subjected to Fast Fourier Transform (FFT). The following harmonic power components were extracted from the magnitude spectrum:

H1 to H4: Power of the first to fourth harmonics in the PPG waveform.

These components represent the periodicity and shape of the waveform, with H1 corresponding to the fundamental frequency and higher-order harmonics (H2–H4) reflecting waveform sharpness and reflection intensity.

2. LF, HF, and LF/HF Ratio

To assess autonomic nervous system activity, we applied pulse rate variability (PRV) analysis using the following procedure:

Pulse Peak Detection: Systolic peaks were detected to construct a series of beat-to-beat intervals (analogous to RR intervals).

Resampling: The interval series was interpolated to 4 Hz using cubic spline interpolation.

Spectral Analysis: Power spectral density was estimated using Welch’s method.

Band Integration: Frequency components were integrated into:

Low Frequency (LF): 0.04–0.15 Hz
High Frequency (HF): 0.15–0.4 Hz

Ratio Calculation: The LF/HF ratio was computed as a marker of sympathovagal balance.

These features—especially LF/HF—reflect autonomic regulation of the cardiovascular system and have been linked to blood pressure modulation.

List of all extracted features grouped by domain. Time-domain parameters include morphological indices, slope measurements, area, and heart rate variability (HRV) metrics. Frequency-domain parameters include harmonic components obtained from fast Fourier transform (FFT) of the PPG waveform and spectral power from interpolated pulse rate variability (PRV). Abbreviations: Ts, systolic duration; Td, diastolic duration; Ss, systolic slope; Ds, diastolic slope; ΔT, time interval between P1 and P2; RMSSD, root mean square of successive differences; SDNN, standard deviation of NN intervals; PNN50, percentage of NN interval differences >50 m; LF, low-frequency power; HF, high-frequency power.

### 2.5 Statistical analysis

All statistical analyses were conducted using *python*, and a two-tailed p value <0.05 was considered statistically significant.

Participants were classified into four groups according to their systolic blood pressure (SBP in mmHg):

1. Group 1: SBP≤120
2. Group 2: 120<SBP≤139
3. Group 3: 140≤SBP≤159
4. Group 4: 160≤SBP

Group Comparison.

All extracted PPG features were summarized as mean ± standard deviation (SD) for each group.

The normality of each variable was assessed using the Shapiro–Wilk test.

For normally distributed variables, one-way ANOVA with *post hoc* Tukey’s test was used to assess between-group differences.

For non-normally distributed features, the Kruskal–Wallis test followed by Dunn’s multiple comparison test was applied.

Effect sizes for non-parametric tests were calculated using epsilon squared (ε^2^), interpreted according to the following thresholds: small 0.01–0.059, medium 0.06–0.139, large ≥0.14.

**FIGURE 5:**
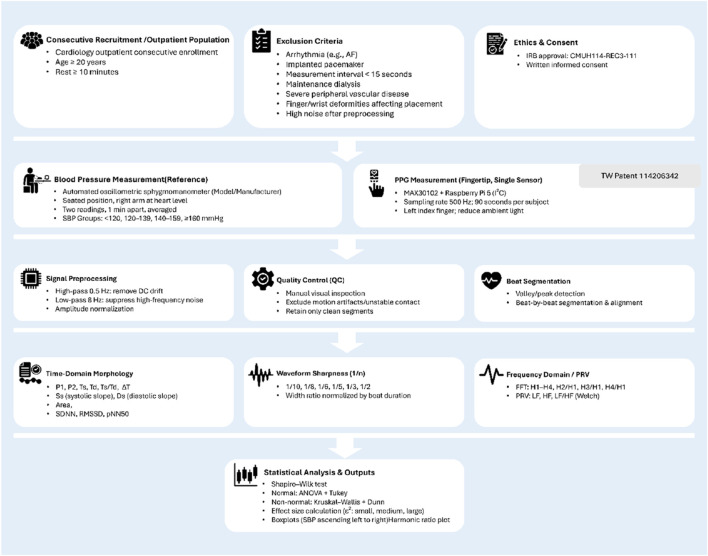
Flowchart of the experimental design and analysis pipeline. Overview of participant recruitment, measurement protocols, signal preprocessing, feature extraction, and statistical analysis steps used in the study. Consecutive adult participants were recruited from cardiology outpatient clinics and underwent both reference blood pressure measurement (automated oscillometric device) and single-site photoplethysmography (PPG) recording using a MAX30102 sensor connected to a Raspberry Pi 5. Signal preprocessing included high-pass and low-pass filtering, amplitude normalization, and manual quality control. Beat-by-beat segmentation allowed extraction of time-domain morphology, waveform sharpness, and frequency-domain/PRV features. Statistical comparisons between SBP groups were performed after normality testing, with results visualized as boxplots. Icons adapted from Flaticon (www.flaticon.com).

All analyses were designed to evaluate whether specific PPG-derived features could differentiate between blood pressure categories and potentially serve as non-invasive markers of hypertensive status. The overall experimental design and analysis pipeline are summarized in Figure 5.

## 3 Results

### 3.1 Participant characteristics (baseline)

#### 3.1.1 Baseline characteristics of the participants

All participants were assigned into four groups based on systolic blood pressure (SBP): ≤120, 121–139, 140–159, and ≥160 mmHg. Baseline characteristics are presented in Tables 2a, 2b. There were no significant differences among SBP groups in the distribution of sex (p = 0.488), height (162.33 ± 8.78 vs. 162.54 ± 9.67 vs. 161.93 ± 8.57 vs. 161.42 ± 9.79 cm, p = 0.818), body weight (67.56 ± 13.44 vs. 66.02 ± 14.32 vs. 67.30 ± 15.29 vs. 71.74 ± 20.16 kg, p = 0.416), or age (64.50 ± 12.79 vs. 62.16 ± 14.07 vs. 65.99 ± 11.72 vs. 66.04 ± 14.52 years, p = 0.119).

**TABLE 2:** Baselinedemographic and anthropometric characteristics of participants across systolic blood pressure (SBP) groups. Continuous variables (Table 2a) are presented as mean ± standard deviation and compared using one-way ANOVA or Kruskal–Wallis test as appropriate. Categorical variables (Table 2b) and clinical parameters (Table 2c) are presented as counts and compared using the chi-squared test. *p*values indicate between-group differences; *p*< 0.05 is considered statistically significant.

Variables	Group				*p*value
	SBP ≤120(n=106)	120<SBP≤139(n=218)	140≤SBP≤159(n=190)	SBP ≥160(n=76)	
(a) Continuous variables					
Height(cm)	162.54 ±9.67	162.33 ± 8.78	161.93 ± 8.57	161.42 ± 9.79	0.818
Body Weight(kg)	66.02 ± 14.32	67.56 ± 13.44	67.30 ± 15.29	71.74 ± 20.16	0.416
BMI(kg)	24.82 ± 4.06	25.52 ± 3.93	25.51 ± 4.83	27.22 ± 5.97	0.049*
Age (years)	62.16 ± 14.07	64.50 ± 12.79	65.99 ± 11.72	66.04 ± 14.52	0.119

Sex	Group				*p*value
	SBP ≤120n=106)	120<SBP≤139 (n=218)	140≤SBP≤159 (n=190)	SBP ≥160 (n=76)	
(b) Categorical variables					
Male	49	96	75	37	
Female	57	122	115	39	0.488

Categorical variables	Group				*p*value
	SBP ≤120	120<SBP≤139	140≤SBP≤159	SBP ≥160	
(c) Clinical parameters					
DM	30.2%	34.9%	40.5%	25.0%	0.073
CAD	50.0%	33.0%	35.3%	26.3%	0.005*
CHF	17.9%	11.9%	7.9%	15.8%	0.059
Thyroid disease	5.7%	1.4%	2.6%	9.2%	0.008*
COPD/Asthma	3.8%	1.4%	1.6%	0.0%	0.240
Dyslipidemia	65.1%	62.8%	57.9%	56.6%	0.648

Continunous variables	SBP ≤120	120<SBP≤139	140≤SBP≤159	SBP ≥160	*p*value
LVEF	58.9 ± 8.3 (n = 88)	59.9 ± 8.3 (n = 175)	60.3 ± 6.9 (n = 148)	59.7 ± 8.0 (n = 55)	0.658
eGFR	82.4 ± 25.7 (n = 100)	77.9 ± 23.0 (n = 206)	75.4 ± 24.3 (n = 181)	73.8 ± 26.6 (n = 75)	0.069
LDL	87.5 ± 33.2 (n = 90)	91.0 ± 29.1 (n = 197)	89.5 ± 35.5 (n = 170)	97.5 ± 31.7 (n = 73)	0.230

* indicates p < 0.05.

However, BMI differed significantly across SBP groups (p = 0.049). The mean ± standard deviation values were 24.82 ± 4.06, 25.52 ± 3.93, 25.51 ± 4.83, and 27.22 ± 5.97 kg/m^2^for the ≤120, 121–139, 140–159, and ≥160 mmHg groups, respectively.

In terms of clinical parameters (Table 2c), the prevalence of CAD differed significantly among SBP groups (50.0% vs. 33.0% vs. 35.3% vs. 26.3%, p = 0.005). Thyroid disease was also more common in the highest SBP group (5.7% vs. 1.4% vs. 2.6% vs. 9.2%, p = 0.008). In contrast, there were no significant differences across SBP groups in the distribution of DM (30.2% vs. 34.9% vs. 40.5% vs. 25.0%, p = 0.073), CHF (17.9% vs. 11.9% vs. 14.7% vs. 15.8%, p = 0.590), COPD/asthma (3.8% vs. 1.4% vs. 1.6% vs. 0.0%, p = 0.240), or dyslipidemia (65.1% vs. 62.8% vs. 57.9% vs. 56.6%, p = 0.648).

Regarding continuous clinical variables, no significant differences were observed among groups for LVEF (58.9% ± 8.3% vs. 59.9% ± 8.3% vs. 60.3% ± 6.9% vs. 59.7% ± 8.0%, p = 0.658), eGFR (82.4 ± 25.7 vs. 77.9 ± 23.0 vs. 75.4 ± 24.3 vs. 73.8 ± 26.6 mL/min/1.73 m^2^, p = 0.069), or LDL cholesterol levels (87.5 ± 33.2 vs. 91.0 ± 29.1 vs. 89.5 ± 35.5 vs. 97.5 ± 31.7 mg/dL, p = 0.230).

### 3.2 Group differences in PPG parameters

#### 3.2.1 Waveform sharpness parameters

Statistical comparisons revealed that six waveform sharpness parameters—1_10, 1_8, 1_6, 1_5, 1_3, and 1_2—differed significantly across the four SBP groups (Kruskal–Wallis test, p < 0.001 for all).

- 1_10: 0.084 ± 0.009 (≤120 mmHg), 0.087 ± 0.011 (121–139 mmHg), 0.090 ± 0.011 (140–159 mmHg), 0.093 ± 0.010 (≥160 mmHg)
- 1_8: 0.094 ± 0.010, 0.098 ± 0.013, 0.101 ± 0.012, 0.104 ± 0.011
- 1_6: 0.109 ± 0.012, 0.113 ± 0.015, 0.117 ± 0.014, 0.121 ± 0.013
- 1_5: 0.120 ± 0.013, 0.124 ± 0.016, 0.129 ± 0.016, 0.133 ± 0.015
- 1_3: 0.158 ± 0.018, 0.165 ± 0.022, 0.171 ± 0.022, 0.176 ± 0.021
- 1_2: 0.203 ± 0.024, 0.212 ± 0.030, 0.219 ± 0.028, 0.226 ± 0.032

**FIGURE 6:**
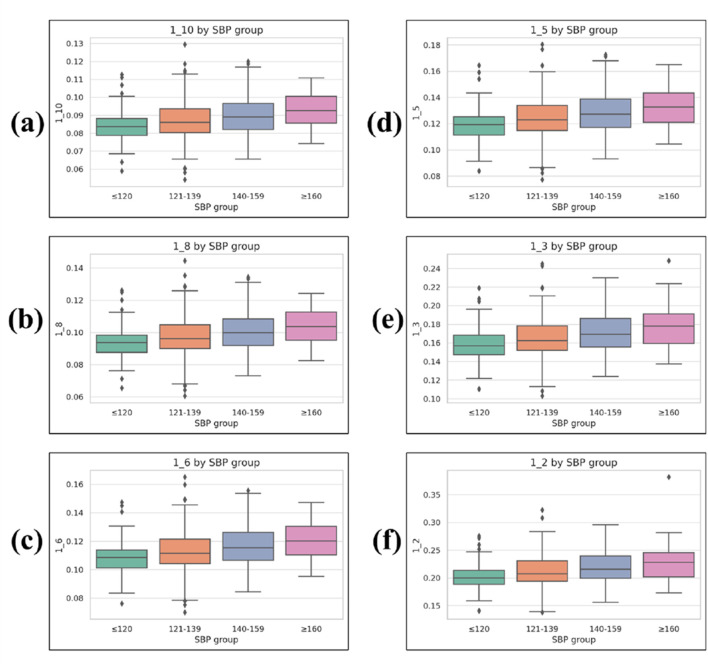
Boxplots of waveform sharpness parameters across systolic blood pressure (SBP) groups. **(a)** 1_10, **(b)** 1_8, **(c)** 1_6, **(d)** 1_5, **(e)** 1_3, and **(f)** 1_2. Each parameter represents the normalized width of the PPG waveform at a given fractional height of the systolic peak (1/n), measured from the left and right intersection points with the target height, and normalized by the total beat duration. Boxplots display median, interquartile range (IQR), whiskers (1.5 × IQR), and outliers.

These results indicate a consistent relationship between increasing SBP level and greater pulse waveform sharpness (Figure 6).

#### 3.2.2 Heart rate variability (HRV) parameters

Among the HRV indices, RMSSD exhibited significant differences across SBP groups (p = 0.012), with values of 31.642 ± 18.273 in the ≤120 mmHg group, 38.215 ± 27.894 in the 121–139 mmHg group, 42.506 ± 35.872 in the 140–159 mmHg group, and 43.391 ± 40.391 in the ≥160 mmHg group.

Similarly, SDNN, reflecting overall heart rate variability, showed values of 111.582 ± 75.421, 122.908 ± 80.216, 127.349 ± 90.157, and 130.485 ± 95.214 for the four groups, respectively, but the difference did not reach statistical significance (p = 0.106).

The PNN50 index, representing the proportion of successive RR intervals differing by more than 50 m, demonstrated significant differences (p = 0.025), with values of 11.770 ± 14.600, 15.832 ± 17.312, 17.245 ± 18.119, and 17.789 ± 18.502 for the ≤120, 121–139, 140–159, and ≥160 mmHg groups, respectively.

#### 3.2.3 Time interval parameters

**FIGURE 7:**
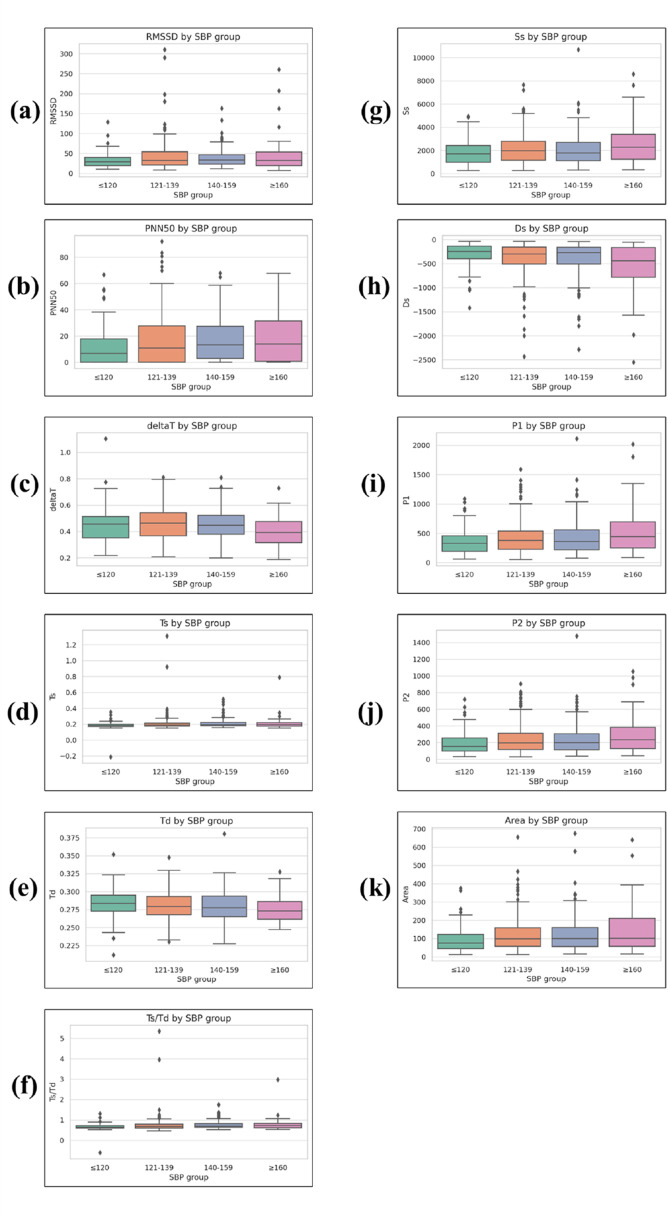
Boxplots of time-domain and morphological PPG parameters across systolic blood pressure (SBP) groups. **(a)** RMSSD, **(b)** PNN50, **(c)** ΔT, **(d)** Ts, **(e)** Td, **(f)** Ts/Td, **(g)** Ss, **(h)** Ds, **(i)** P1, **(j)** P2, and **(k)** Area. RMSSD and PNN50 are heart rate variability (HRV) indices. Ts, Td, Ts/Td, and ΔT are systolic/diastolic time intervals. Ss and Ds represent systolic and diastolic slopes, respectively. P1 is the amplitude of the primary systolic peak, P2 is the reflected wave peak amplitude after the dicrotic notch, and Area represents the integrated waveform area over the cardiac cycle. Boxplots display median, interquartile range (IQR), whiskers (1.5 × IQR), and outliers.

Time-domain markers related to systolic and diastolic intervals exhibited statistically significant variation across SBP groups (Figure 7).

The total duration from systolic peak to diastolic peak (deltaT) showed a significant group effect (p = 0.002), with values of 0.458 ± 0.118 in the ≤120 mmHg group, 0.431 ± 0.115 in the 121–139 mmHg group, 0.430 ± 0.104 in the 140–159 mmHg group, and 0.399 ± 0.105 in the ≥160 mmHg group.

The systolic duration (Ts) increased slightly across SBP levels (p < 0.001), measured at 0.371 ± 0.089, 0.397 ± 0.082, 0.402 ± 0.076, and 0.407 ± 0.083 for the four ascending SBP groups.

The diastolic duration (Td) decreased modestly (p = 0.004), with values of 0.574 ± 0.143, 0.527 ± 0.132, 0.527 ± 0.118, and 0.508 ± 0.118 for the respective SBP groups.

This led to a significant difference in the Ts/Td ratio (p < 0.001), with values of 0.664 ± 0.172, 0.765 ± 0.296, 0.781 ± 0.270, and 0.802 ± 0.302 in the ≤120, 121–139, 140–159, and ≥160 mmHg groups, respectively.

#### 3.2.4 Waveform peak parameters

Peak-related parameters also demonstrated significant group-level differences.

The first systolic peak (P1) showed increasing mean values across SBP categories (p = 0.011), with values of 355.196 ± 216.961 in the ≤120 mmHg group, 450.782 ± 302.154 in the 121–139 mmHg group, 510.348 ± 364.214 in the 140–159 mmHg group, and 539.606 ± 394.371 in the ≥160 mmHg group.

A similar pattern was observed for the secondary peak (P2) (p = 0.012), with mean values of 191.490 ± 135.458, 244.587 ± 176.303, 281.062 ± 209.411, and 293.040 ± 224.599 for the ≤120, 121–139, 140–159, and ≥160 mmHg groups, respectively.

#### 3.2.5 Slope and area parameters

Slope-related metrics and waveform area also varied significantly with SBP levels.

The systolic slope (Ss) increased significantly among groups (p = 0.021), with mean values of 1828.996 ± 1081.809 in the ≤120 mmHg group, 2256.317 ± 1482.212 in the 121–139 mmHg group, 2365.774 ± 1615.693 in the 140–159 mmHg group, and 2621.842 ± 1801.847 in the ≥160 mmHg group.

Conversely, the diastolic slope (Ds) became more negative with higher SBP levels (p = 0.006), measured at −312.309 ± 249.905, −451.672 ± 353.824, −533.245 ± 451.512, and −566.816 ± 503.391 for the ≤120, 121–139, 140–159, and ≥160 mmHg groups, respectively.

#### 3.2.6 Area parameter

The Area parameter, representing the integrated area under the PPG waveform, showed significant variation across SBP categories (p = 0.012). Mean values were 40.933 ± 10.563 in the ≤120 mmHg group, 44.832 ± 15.217 in the 121–139 mmHg group, 48.219 ± 19.526 in the 140–159 mmHg group, and 49.278 ± 24.044 in the ≥160 mmHg group, indicating a general tendency for larger waveform areas at higher SBP levels.

#### 3.2.7 Frequency-domain parameters

**FIGURE 8:**
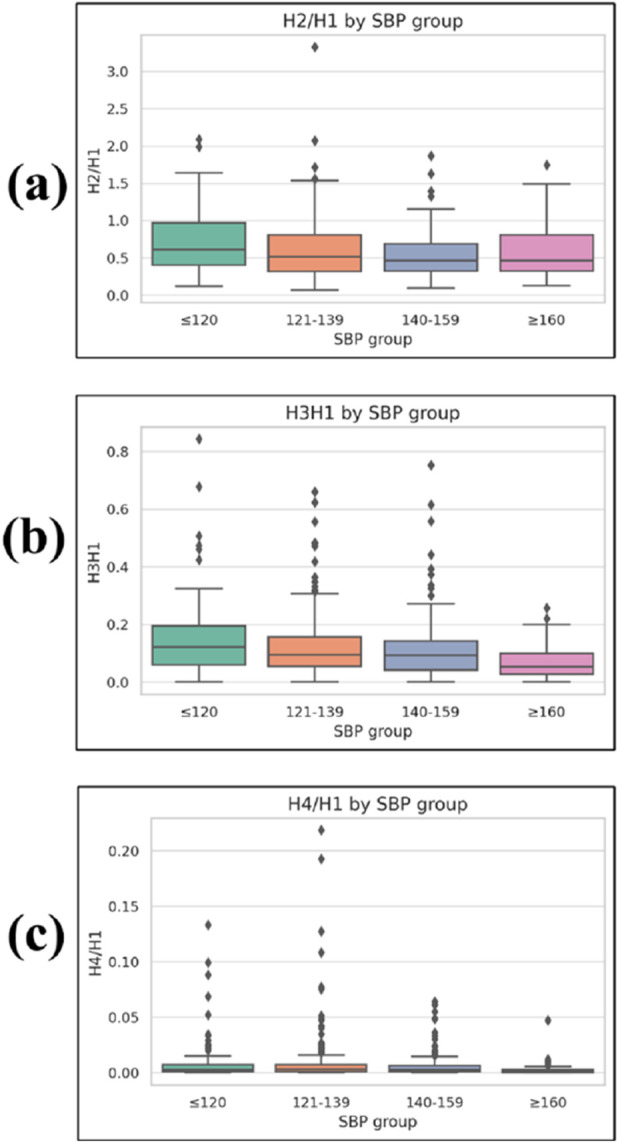
Boxplots of frequency-domain harmonic ratios across systolic blood pressure (SBP) groups. **(a)** H2/H1, **(b)** H3/H1, and **(c)** H4/H1, where H1–H4 represent the first to fourth harmonic amplitudes from the PPG waveform Fourier spectrum, and ratios are normalized to H1. Lower harmonic ratios indicate a greater concentration of spectral energy in the fundamental frequency. Boxplots display median, interquartile range (IQR), whiskers (1.5 × IQR), and outliers.

Three frequency-domain parameters based on harmonic decomposition of the pulse waveform showed statistically significant differences across SBP groups (Figure 8).

The H2/H1 ratio was 0.711 ± 0.411 in the ≤120 mmHg group, 0.667 ± 0.389 in the 121–139 mmHg group, 0.534 ± 0.299 in the 140–159 mmHg group, and 0.598 ± 0.347 in the ≥160 mmHg group, with a significant group effect (p = 0.006).

The H3/H1 ratio was 0.147 ± 0.133, 0.099 ± 0.092, 0.086 ± 0.067, and 0.069 ± 0.058 for the ≤120, 121–139, 140–159, and ≥160 mmHg groups, respectively, showing a significant difference among groups (p < 0.001).

The H4/H1 ratio was 0.009 ± 0.020, 0.009 ± 0.024, 0.006 ± 0.010, and 0.002 ± 0.006 across the four SBP groups in ascending order, also demonstrating a significant difference (p < 0.001).

Other spectral features, including low-frequency power (LF; 0.00025 ± 0.00058, 0.00012 ± 0.00034, 0.00013 ± 0.00034, and 0.00018 ± 0.00037), high-frequency power (HF; 0.00143 ± 0.00307, 0.00080 ± 0.00204, 0.00053 ± 0.00122, and 0.00083 ± 0.00178), and the LF/HF ratio (0.179 ± 0.271, 0.183 ± 0.290, 0.320 ± 0.643, and 0.315 ± 0.430), did not show statistically significant differences among groups (p = 0.105, 0.060, and 0.916, respectively).

#### 3.2.8 Effect size analysis of significant features

To further evaluate the magnitude of group differences, we calculated effect sizes (ε^2^) for all PPG-derived features that showed statistically significant differences across SBP categories in the previous subsections. Effect size classification was based on Cohen’s thresholds (small: 0.01–0.059, medium: 0.06–0.139, large: ≥0.14). Table 3 summarizes each significant feature’s category, p-value, ε^2^, and effect size classification.

**TABLE 3:** Effect sizes (ε^2^) and p-values for photoplethysmography (PPG) features with significant differences across systolic blood pressure (SBP) groups. The table lists each significant feature’s category, p-value, epsilon squared (ε^2^), and effect-size classification. Effect-size thresholds follow Cohen’s convention: small 0.01–0.059, medium 0.06–0.139, large ≥0.14. Abbreviations: PPG, photoplethysmography; PRV, pulse rate variability.

Feature	Category	p-value	$\varepsilon^{2}$	Effect size
1_10	Waveform sharpness	<0.001	0.060	Medium
1_8	Waveform sharpness	<0.001	0.060	Medium
1_5	Waveform sharpness	<0.001	0.060	Medium
1_6	Waveform sharpness	<0.001	0.060	Medium
1_3	Waveform sharpness	<0.001	0.060	Medium
1_2	Waveform sharpness	<0.001	0.055	Small
H3/H1	Frequency-domain	<0.001	0.047	Small
H4/H1	Frequency-domain	<0.001	0.032	Small
Ts/Td	Time interval	<0.001	0.031	Small
Ts	Time interval	<0.001	0.028	Small
deltaT	Time interval	0.002	0.020	Small
Td	Time interval	0.004	0.018	Small
Ds	Slope	0.006	0.016	Small
H2/H1	Frequency-domain	0.005	0.016	Small
P1	Waveform peak	0.011	0.014	Small
RMSSD	HRV	0.012	0.014	Small
Area	Area	0.012	0.014	Small
P2	Waveform peak	0.012	0.014	Small
Ss	Slope	0.021	0.012	Small
PNN50	HRV	0.025	0.011	Small

## 4 Discussion

### 4.1 Hemodynamic basis of PPG morphology and blood pressure

Photoplethysmographic (PPG) waveforms recorded at peripheral sites are shaped by fundamental hemodynamic principles that link arterial morphology to blood pressure (Matti Huotari et al., 2011; Yousef et al., 2012; Karimpour et al., 2023). According to the relationship BP = CO × PVR, in individuals with stable cardiac output, elevated blood pressure primarily reflects an increase in peripheral vascular resistance (PVR). When the left ventricle ejects blood, it generates a forward-traveling pressure wave that propagates through the arterial tree. Along its path, the wave encounters sites of impedance—such as arteriolar branching points or microvascular beds—where part of the energy is reflected. The superposition of this reflected wave with the forward wave produces the composite peripheral pulse waveform observed in arteries such as the radial or femoral artery (Hashimoto and Ito, 2010).

In the PPG signal, this interaction often results in a tricrotic configuration, with distinct morphological components including the dicrotic notch, a marker of the transition from systole to diastole and an indicator of ventricular–arterial coupling (Hashimoto and Ito, 2010). The timing and magnitude of these waveform components are governed by arterial stiffness and compliance, as described by the Moens–Korteweg equation, which relates pulse wave velocity (PWV) to vessel elasticity, wall thickness, blood density, and internal diameter (von Wowern et al., 2017; Wu et al., 2022).

In hypertension, increased arterial stiffness accelerates PWV, causing the reflected wave to return earlier—often during systole rather than diastole (Chen et al., 2005). This early wave superposition elevates central systolic pressure, augments pulse pressure, and modifies the waveform’s amplitude, slope, and width. In addition, higher PVR reduces the Windkessel buffering effect, amplifying both the forward and reflected wave peaks. Consequently, the morphological features of the PPG waveform serve as physiologically meaningful indicators of vascular load, offering non-invasive insight into the hemodynamic alterations associated with elevated blood pressure (Szoltysek-Boldys et al., 2024).

### 4.2 Review of existing theoretical frameworks

The relationship between pulse wave velocity (PWV) and blood pressure has been recognized for more than a century, with early studies in the late 19th century already reporting a strong positive correlation (Hellqvistet al. 2024). The Moens–Korteweg and Bramwell–Hill equations established the biomechanical foundation for PWV-based estimation of arterial pressure, linking vessel stiffness, wall thickness, and lumen diameter to the speed of pulse propagation. Building on these principles, pulse transit time (PTT)—the interval between proximal and distal pulse waveforms—has been widely explored as a noninvasive and cuffless approach for blood pressure estimation (Ghoshet al. 2023). However, conventional PTT measurements require at least two synchronized sensors (e.g., ECG and PPG), which constrains their feasibility for continuous, unobtrusive monitoring in daily-life settings (Witteveen et al., 2025).

In recent years, research emphasis has shifted toward pulse waveform analysis (PWA), in which morphological features extracted from a single peripheral waveform are analyzed to infer vascular status and hemodynamic load (Pankaj et al., 2023). Time-domain parameters such as amplitude, area, systolic and diastolic phase durations (Ts and Td), as well as indices of waveform sharpness and slope, have shown significant associations with systolic blood pressure in both experimental and clinical studies (Alian et al., 2016). This paradigm reduces hardware complexity while potentially capturing richer physiological information beyond transit time alone. The present study aligns with this evolution by focusing on the extraction of novel, clinically interpretable PPG features from a single-sensor configuration, enabling practical, noninvasive assessment of blood pressure-related vascular changes in real-world settings (Perdereau et al., 2025).

### 4.3 Spectral domain analysis and harmonic indicators

In this study, we applied Fourier decomposition to PPG signals to quantify the relative energy distribution across harmonic components, with a particular focus on higher-order harmonic amplitude ratios (e.g., H2/H1, H3/H1, H4/H1) (Wu et al., 2021). These harmonic ratios provide a compact representation of waveform complexity and have direct physiological relevance to vascular properties (Park et al., 2023).

From a hemodynamic perspective, elevated blood pressure is accompanied by increased arterial stiffness, which accelerates pulse wave velocity and results in the earlier return of reflected waves from the peripher (Hsu et al., 2014). This altered wave reflection timing modifies the pulse contour by steepening the systolic upstroke, shortening diastolic decay, and diminishing or eliminating the dicrotic wave. Such morphological simplification reduces the complexity of the waveform profile, with diminished diastolic features, thereby concentrating spectral energy in the fundamental frequency while reducing the relative contribution of higher-order harmonics (Liao et al., 2020).

The observed inverse relationship between harmonic ratios and systolic blood pressure in our cohort is consistent with prior studies showing that attenuation of higher harmonics reflects reduced arterial compliance, impaired microvascular elasticity, and altered wave propagation dynamics (Chang et al., 2016). This spectral shift may serve as a sensitive marker of hypertension-related vascular changes, offering potential utility for noninvasive vascular load assessment and risk stratification in both clinical and wearable device applications (Hsiu et al., 2012).

### 4.4 Discussion of significant PPG parameters by category

#### 4.4.1 Waveform sharpness parameters

In this study, all six waveform sharpness parameters (1_10, 1_8, 1_6, 1_5, 1_3, and 1_2) demonstrated statistically significant differences across SBP groups. These indices quantify the relative width of the PPG waveform at specific fractional heights of the systolic peak, normalized to the total beat duration. Higher values indicate a broader contour, whereas lower values indicate a steeper, sharper upstroke.

Physiologically, waveform sharpness reflects the interaction between the forward pressure wave generated by left ventricular contraction and the reflected wave returning from peripheral sites. In individuals with elevated SBP, increased arterial stiffness reduces vascular compliance and accelerates pulse wave velocity, leading to earlier arrival of the reflected wave. This earlier wave reflection merges with the forward wave during systole, modifying the upstroke morphology. While earlier reflection is often associated with a narrower systolic peak, prior studies using both PPG and invasive arterial pressure waveforms have demonstrated that in stiff arteries, wave summation may also broaden the waveform in the mid-to-upper systolic segment due to augmented reflection (Millasseau et al., 2002).

#### 4.4.2 Heart rate variability (HRV) parameters

Statistically significant differences were seen among three HRV indices compared. RMSSD increased from 31.642 ± 18.273 m in the ≤120 mmHg group to 43.391 ± 40.391 m in the ≥160 mmHg group (p = 0.012). These changes were statistically meaningful. The same was true of PNN50, which rose from 11.770% ± 14.600% to 17.789% ± 18.502% in the two body-weight categories (p = 0.025).

In terms of absolute increase and variability, this modest change in HRV indices is opposite the trend seen in most HRV studies based on ECG data: hypertension lowers vagally mediated HRV, as we saw right down at the beginning of this paper when examining patients chronically subjected to high blood pressure conditions or in which they had just recently entered such circumstances. These divergences may be attributable to both physiological and technical causes.

ECG-derived HRV measures the degree of modulation by the human autonomic nervous system of the heart’s primary pacemaker, the sinoatrial node. But PRV also records statistical effects resulting from endothelial function, arterial stiffness, and an accruement of earlier-reflected waves in the pulse pressure wave. When individuals with hypertension have increased stiffness in their arteries, these factors will together boost beat-to-beat variability in the waveform or contour of peripheral pulse waves, potentially giving an inflated reading for RMSSD or PNN50, though central autonomic regulation has been compromised physiologically.

Our results are in line with recent reports showing that PRV can provide excessive estimates of HRV when the vasculature becomes part of the system of signals or when there are patients with unstable peripheral perfusion states. It is crucial to place these figures on a vital foundation by interpreting them not as stand-alone data but within a larger framework than just the heart (Javorka et al., 2024).

#### 4.4.3 Time interval parameters

In the study, all timing parameters, three of which were examined, showed significant differences in system blood pressure (SBP) category compared to healthy people. Groups with higher readings were generally longer than those averaging only 120 mmHg: with increasing SBP, Ts lengthened; Td became briefer, and in turn, the Ts/Td ratio increased, all resulting from this shift. Now a greater portion of the heart is occupied by systole than before in patients with hypertension.

From a physiological point of view, longer Ts in hypertension reflects a higher left ventricular afterload caused by resistance to blood flow and less compliant arteries in your body (Rosenwasser et al., 2014). In a way, the larger proportion of the cardiac cycle taken up by systole as these people get older and sicker is an interesting irony of this condition. Stiffened central arteries cause the forward wave of pressure to be transmitted more quickly, and it comes back earlier in the heart’s periodic cycle than normal. When this reflected wave happens during very late systole instead of during diastole, central aortic systolic pressure actually rises (Wilkinson et al., 2000). At the same time, the ejection phase prolongs and diastolic filling—and thus coronary perfusion—are compressed due to the reason that increasingly less time remains for diastole. When this reflected wave overlaps with late systole rather than diastole, it effectively increases central aortic systolic pressure, prolongs the ejection phase, and reduces the time available for diastolic filling and coronary perfusion (Wilkinson et al., 2000).

It was observed that shortened Td, in turn, provides evidence supporting the mechanism just outlined since diastole is the phase where the coronary arteries receive the lion’s share of their blood flow. In hypertension, a decrease in Td may further increase the risk for myocardial ischemic posterior wall abnormalities, especially in patients with combined left ventricular hypertrophy and/or coronary artery disease (Carlini et al., 2024).

The delta T parameter, representing the time interval between P1 (the primary systolic peak) and P2 (the secondary peak or inflection point), also showed a significant decrease with higher SBP. This is consistent with the well-established principle that arterial stiffening makes pulse wave travel time shorter and so causes an earlier return of the reflected wave (Gurovich et al., 2009); the early reflection at central A. When this reflection occurs so early that in advanced arterial stiffness it comes to merge with the forward systolic wave, central aortic systolic pressure goes up again, pulse pressure takes on an even greater burden of work being added to your heart, and left ventricular workloads increase.

To some extent, this is like a pump which is unable to expand properly from touching its target against the wall—under such conditions the only way out for things gotten inside will either have to go up above the top or become stuck inside (the author’s italics). Earlier than normal reflection can lead to large variation in blood pressure (BP) in elderly people who have developed advanced arterial stiffness. As the pulse travels down the aorta, it reaches farther areas and thus has to flow through enlarging vessels. This slows its rate of return back toward the heart considerably.

By supporting previous findings, our study has shown that the P1-P2 interval, systolic duration, and systolic-to-diastolic ratio obtained from pulse wave analysis, all correlate well with carotid-femoral pulse wave velocity (cfPWV) and augmentation index (AIx), both of which are recognized markers of arterial stiffness and independent predictors of cardiovascular risk (Rosenwasser et al., 2014). Our data provide further support for the notion that PPG-derived timing parameters can provide insight into the dynamic relationship between ventricular ejection and vascular load, in addition to serving as convenient noninvasive measures of hypertensive vascular remodeling and hemodynamic burden.

#### 4.4.4 Waveform peak and amplitude parameters

Both the systolic peak (P1) and diastolic peak (P2) amplitudes measured with reference to baseline were higher in participants with high resting SBP in this study.

P1: Left ventricular pressure wave velocity of forward-travelling (generated by left ventricular ejection).

P2: Reflected waves as return from peripheral resistance sites.

These authors reasoned that the observed increase in P1 inner amplitude in hypertensives could easily be explained by a large stroke volume being ejected into a less compliant arterial system combined with an insufficient filling of Windkessel capacitors. However, a greater stiffness of the arterial tree results in higher ventricular pressure, which then leads to a larger instantaneous rise in pressure from the same amount of blood ejected by the heart and raises the systolic peak of the PPG wave (Hersant et al., 2025).

An increased P2 amplitude in the hypertensive groups indicates more prominent wave reflection from the periphery, increasing the return of a proportion of the reflected waves some distance upstream before systole (Betge et al., 2017). This worsens pulsus load on the wall of the artery by not only enhancing the systolic pressure in central but also increasing the pulse pressures. P2 or a higher augmentation index derived from PPG or tonometry is strongly correlated with increased peripheral vascular resistance and central aortic stiffness, as shown in previous studies (Korolev et al., 2023), etiologies which are common in hypertension.

On the clinical side, amplitude-based view, similarity, and spectral solidity are more intuitive to see. The intuitive physiological interpretation of these PRV metrics and their extractability from a single-site PPG signal position them as strong candidates for incorporation into user-oriented screening tools or wearable devices designed to diagnose individuals with hypertension on the one hand, and establish indices of vascular function on the other.

#### 4.4.5 Slope and area parameters

In our study, both systolic slope (Ss) and diastolic slope (Ds) demonstrated significant associations with SBP across all groups. Higher SBP was characterized by a steeper Ss, reflecting a more rapid systolic upstroke (O'Rourke et al., 2002), and a more negative Ds, indicating a sharper and more pronounced diastolic decline (Hashimoto and Ito, 2017). These alterations of the slopes are in keeping with central pulse wave velocity and arterial stiffness, respectively with faster forward pressure wave transmission in conjunction with a smaller capacitative Windkessel volume during diastole. This was a summary value for all values combined within the pulse and integrated both amplitude and temporal components of the pulse.

Given that hypertensive persons may have an increased stroke volume and higher peripheral pressure load, this phenomenon is perhaps not surprising. Previous studies have indicated that the augmentation of wave reflection and increased afterload can cause a wider and higher area waveform mainly for patients with systolic hypertension (Cauwenberghs et al., 2019).

Altogether, the higher Ss, lower Ds, and increased area of loops provide a characteristic pattern of an elevated vascular load and impaired arterial compliance in hypertension. Interestingly, this is consistent with hemodynamic theories showing that vascular stiffening may be due to a faster systolic rise and steeper diastolic decay of the arterial pressure wave resulting in an increased energy content.

#### 4.4.6 Frequency-domain harmonic parameters

Three harmonic amplitude ratios (H2/H1, H3/H1, and H4/H1) showed significant group-level differences in the present study, with all showing a stepwise decrement towards lower SBP. This trend suggests that the overall pulse waveform relies less on higher-order harmonics as a fraction of harmonic energy in hypertensives. This attenuation can be physiologically explained by a simplification of the pulse contour, with respect to waveform narrowing and dicrotic feature blunting or abolition. These effects are classical consequences of the resulting altered wave reflection retardation caused by elevated arterial stiffness and decreased vascular compliance (O'Rourke, 2009). The increase in arterial stiffness causes the reflected pressure wave from peripheral sites to return at an earlier point, partially merging with the forward wave during late systole. This earlier overlap reduces the amplitude and clarity of secondary peaks, thereby attenuating the energy content in the higher-frequency spectrum (Song et al., 2025).

The importance of harmonic attenuation in vascular pathophysiology has been highlighted in previous studies. Milkovich et al. introduced the concept of harmonic distortion (HD) in blood pressure waveforms, showing that HD decreases linearly with increasing SBP and measures of arterial stiffness such as pulse wave velocity (PWV). This finding parallels our observation that harmonic ratios decline with elevated vascular load. Furthermore, harmonic analysis theory, including the so-called “resonance theory” of peripheral circulation, posits that vascular beds act as frequency-selective resonators. In this framework, each harmonic component of the PPG signal carries information on specific vascular segments and their elasticity properties. Suppression of the second and higher harmonics has been reported in older and hypertensive populations, reflecting impaired arterial compliance, altered wave propagation, and reduced microvascular elasticity (Rensma et al., 2020).

Our results are in agreement with these previous findings and suggest that, free from the Fourier reconstruction of BP oscillations, no-dimensional measures derived directly from waveform morphologies such as harmonic ratios may be employed to provide an elegant solution for neural models. Our present study indicated no significant difference in traditional pulse rate variability (PRV) spectral indices—LF, HF, or LF/HF—between groups of SBP. This indicates that autonomic balance measures, while useful in other conditions, may not be as sensitive to vasculature changes driven by SBP. In contrast, harmonic amplitude ratios provide a direct connection to mechanical and structural properties of the arterial tree, which could give better discrimination for the ability to stratify risk in hypertension from both a clinical and wearable perspective.

### 4.5 Clinical relevance and future implementation

Despite being a leading and most easily modifiable cardiovascular risk factor of all, hypertension is underdiagnosed in almost half of these patients worldwide, as was emphasized by the recent joint statement by the World Health Organization and The Lancet Global Health. An important element in optimizing hypertension control is then the need for accurate, easily obtained, and noninvasive blood pressure assessment to this end and to balance prolonged under-perfusion with the risks associated with aggressive overtreatment.

We found a variety of PPG-derived morphology and SFA parameters—systolic and diastolic slopes, waveform area, peak amplitude, and harmonic amplitude ratios—that were significantly associated with SBP in our study. The derivation of vascular load and arterial stiffness from these parameters results in a solution that is physiologically interpretable and can serve as a fingerprint for vascular load and arterial stiffness through time-domain as well as frequency-domain information. More specifically, the trend observed in significantly reduced higher-order harmonics ratios by SBP demonstrates that it provides a new spectral index of an abnormal wave reflection process together with decreased vascular compliance. Our strategy benefits from utilizing these functionalities, allowing the detection of more nuanced hemodynamic alterations than what can be observed with traditional heart rate variability measures and increasing our sensitivity to hypertension-related vascular changes.

Our method has the distinction from pulse transit time or pulse wave velocity methods by its single-sensor acquisition design to avoid synchronizing multiple sensors. In addition to reduced hardware costs and setup complexity, this simplification will help lower the adoption threshold for a variety of clinical and community settings. In terms of hospital settings, a single-sensor PPG device can be easily implemented and does not disrupt the flow of work in outpatient clinics, inpatient wards, or emergency departments. It is both portable and requires minimal operator training, allowing for wide-scale hypertension screening in primary care and rural clinics. The integration with smartphones or wearable devices allows for continuous, non-invasive monitoring of vascular health at home, providing a long-term screening assist for hypertension diagnosis and personalized management.

This has been confirmed in several large-scale randomized controlled trials demonstrating that a reduction of SBP by about 10 mmHg reduces the incidence of major CVD events. For example, the Systolic Blood Pressure Intervention Trial (SPRINT) rejected the null hypothesis of no benefit for intensive SBP reduction on significant reductions in cardiovascular risk were confirmed across a range of community-dwelling older adults.

Jamshidian et al. (2025) and chronic kidney disease followed by dialysis (Kurella Tamura et al., 2025). Once again, just like in the LIFE study where subjects were hypertensive, they noticed that antihypertensive therapy also led to a significant reduction of stroke, permitting myocardial infarction, cv death (Dahlof et al., 2002).

Detecting these vascular changes earlier, before symptoms begin and manifest as complications, helps in reducing cardiovascular risk, therefore. By virtue of its noise-resistant design, the low-cost and single-sensor PPG system developed in this work (and protected under Taiwan Patent No. 114206342) represents a practical approach for massive on-site hypertension screening. This has the potential, when incorporated in wearables or other mobile health technologies, to increase hypertension diagnosis rates, allow longitudinal vascular monitoring, and ultimately decrease the burden of cardiovascular disease at an individual as well as population level.

### 4.6 Key points

- Multiple PPG-derived morphological and spectral parameters, including waveform sharpness indices, systolic/diastolic slopes, and harmonic ratios, showed significant associations with systolic blood pressure categories.
- Timing indices such as prolonged systolic duration, shortened diastolic duration, and decreased P1–P2 interval reflected the hemodynamic impact of arterial stiffness and wave reflection.
- Higher-order harmonic amplitude ratios (H2/H1, H3/H1, H4/H1) decreased with increasing SBP, providing a novel spectral marker of impaired vascular compliance.
- In contrast to ECG-derived HRV, PRV indices (RMSSD, PNN50) increased with higher SBP, likely reflecting vascular stiffness effects on pulse wave variability.
- A single, low-cost, and noise-resistant PPG sensor enabled extraction of physiologically interpretable indices relevant to vascular load and arterial stiffness, supporting practical applications for non-invasive hypertension screening and monitoring.

### 4.7 Limitations

This study has several limitations. First, although multiple PPG-derived indicators stood out with statistical significance for SBP, effect sizes tended to remain small (ε^2^ < 0.06) and may restrict clinical value. Second, possible confounders such as diabetes, tobacco smoking, pharmaceutical therapy, and lipid panel failed to be adequately adjusted and residual confounding is possible. Third, although artifacts were deleted manually, no quantitative signal quality index (SQI) was applied, and reproducibility is constrained. Fourth, PRV-based HRV indicators (RMSSD and PNN50) increased with SBP, contrary to most ECG-based HRV studies and warrants future investigation. In addition, clinical markers such as CAD and thyroid disease differed between SBP groups and suggest comorbid conditions may influence hemodynamic responses and introduce heterogeneity in PPG measurements, yet also provide future disease-specific analysis guidelines. Moreover, as all participants were consecutively recruited from a cardiology outpatient department, the study sample may fail to generalize to the general population. Patients attending cardiovascular clinics are more likely to have comorbid conditions, receive prescription pharmaceutical therapy, and show higher baseline cardiovascular risk and may overstate correlations between PPG-derived indicators and SBP. This selection bias limits generalizability and future studies including community-based samples or populational samples are required for confirmation. Finally, gold-standard validation using vascular indices such as cfPWV or AIx was not performed and must be addressed in future studies.

## 5 Conclusion

This clinical validation study demonstrated that multiple photoplethysmography (PPG) morphological and spectral parameters, in particular waveform sharpness indices and higher-order harmonic ratios, were significantly associated with systolic blood pressure categories. These findings highlight the physiological relevance of PPG-derived features and establish the feasibility of using a single, low-cost, and noise-resistant sensor to provide clinically meaningful information on vascular load and arterial stiffness.

## Data Availability

The raw data supporting the conclusions of this article will be made available by the authors, without undue reservation.
