# Supplementary material for: Clinical validation of comprehensive time- and frequency-domain photoplethysmography features from a single-sensor system for non-invasive assessment of vascular load and systolic blood pressure
Source: Front Physiol. 2025 Oct 29;16:1695391. doi: 10.3389/fphys.2025.1695391 (PMC12605234; doi:10.3389/fphys.2025.1695391)
Supplement: Supplementary file 1 [file DataSheet1.pdf]

TableS1.

PPG features	median	Q1	Q3	min	max
1_10	0.08681	0.08085	0.09479	0.05408	0.12948
1_8	0.09711	0.09042	0.10621	0.06058	0.1445
1_6	0.11274	0.10458	0.1232	0.07003	0.16512
1_5	0.1239	0.11518	0.13601	0.0773	0.18037
1_3	0.16337	0.15268	0.18108	0.10302	0.24831
1_2	0.20969	0.19495	0.23451	0.13813	0.38181
deltaT	0.44473	0.36301	0.5221	0.18633	1.10383
P1	362.09	220.108	556.488	52.2769	2111.27
P2	188.585	110.876	310.141	27.7733	1478.54
pulse rate	76.128	67.5806	85.3516	50.2494	118.355
Ts	0.19031	0.17435	0.21531	-0.2117	1.30933
Td	0.27887	0.26682	0.29287	0.21184	0.3804
Ts/Td	0.68166	0.61648	0.79554	-0.6019	5.34968
Ss	1889.16	1102.29	2746.86	268.756	10692.2
Ds	-291.09	-507.91	-147.34	-2551.6	-36.99
Area	95.2419	53.8372	155.656	12.1227	675.288
H2/H1	0.50754	0.32736	0.79494	0.06922	3.32646
H3H1	0.0937	0.04342	0.15086	0.00014	0.84289
H4/H1	0.00239	0.00063	0.00617	2.2E-07	0.2186
SDNN	25.8789	18.4982	37.0178	5.75482	280.48
RMSSD	31.5765	21.5145	48.0463	7.26255	310.341
PNN50	10.5263	0	26.3158	0	92
LF	6.1E-05	2.5E-05	0.00015	5.1E-07	0.03757
HF	0.00014	6.3E-05	0.00038	2.1E-06	0.03365
LF/ HF	0.44099	0.22081	0.88555	0.01634	6.75421
